# Physical Activities and Learning Experience of Higher Education Students: Mediating Role of Quality of Life and Physical Self-Esteem

**DOI:** 10.3390/ijerph192013417

**Published:** 2022-10-17

**Authors:** Mohamed A. Zayed, Ibrahim A. Elshaer

**Affiliations:** 1 Deanship of Student Affairs, King Faisal University, Al-Hassa 31982, Saudi Arabia; 2 Department of Fitness, Gymnastics and Sports Show, Faculty of Physical Education Alexandria University, Alexandria 21625, Egypt; 3 Management Department, College of Business Administration, King Faisal University, Al-Hassa 31982, Saudi Arabia; 4 Faculty of Tourism and Hotel Management, Suez Canal University, Ismailia 41522, Egypt

**Keywords:** quality of life, physical activities, physical self-esteem, learning experience, SmartPLS4, higher education student

## Abstract

The impacts of physical activities (PAs) on psychological and physical health consequences have been examined in both nonclinical and clinical contexts. Although PAs’ influences on physical consequences are regularly beneficial, the psychological positive impacts are less well-understood. This study investigates the effects of PA on physical consequences (i.e., physical self-esteem) and psychological and cognitive consequences (i.e., quality of life and learning experience). PA has been found to improve quality of life (QOL), learning experience (LP), and physical self-esteem. Mixed findings from prior studies suggested that the relationship between these variables might be direct or indirect. Data were collected via a self-administrated questionnaire from 510 higher education students in different Kingdom of Saudi Arabia (KSA) universities. The collected data were analyzed by structural equation modeling (SEM) and the SmartPLS 4 program. The SEM results show that PAs directly help in enhancing the student learning experience. The results support the mediating role of student physical self-esteem and quality of life in the relationship between physical activities and learning experience. Several theoretical and practical implications were elaborated on and discussed, along with limitations and further research opportunities.

## 1. Introduction

Despite their similarities, exercise and physical activity (PA) are not the same things. PA refers to any motion of the body that is generated by the skeletal muscles and results in an increase in the amount of energy expended over the baseline rate; the form of physical activity known as exercise is a subset of PA that includes the characteristics of planning, structure, repetitiveness, and purposeful endeavoring to achieve physical fitness. All these exercises and PA are normally assessed in kilocalories, and PA generates developments in physical fitness as PA frequency, length of time, and intensity increase [1,2]. According to the research on the rate of PA among adult people in 28 European countries, 59.1% of participants were physically active, which was accomplished through vigorous exercise [3]. In 2020, 67% of people over the age of six were involved in fitness sports, according to the PA Council’s overview report [4,5,6]. PA contributes to sustaining excellent health and the enhancement of quality of life (QOL) and physical self-esteem (PSE) [7,8].

Both “mental health” and “quality of life” are often used as umbrella concepts to refer to mental, psychological, affective, and cognitive factors that either enhance or impair a person’s functioning. Therefore, the psychological, subjective, and overall quality of one’s life have been singled out as major factors that contribute to one’s mental health. This viewpoint is in line with the theoretical definitions of quality of life and wellbeing, both of which place an emphasis on the absence of mental and physical illness [7]. The psychological models and theories of quality of life emphasize, in a corresponding manner, that quality of life is related to having optimal experiences as well as optimal functioning of both the body and the mind [6]. As a result, previous research (including meta-analyses and reviews) looked into the connection between physical activity and its consequences, which included mental health issues such as depression and anxiety, as well as mood and affect, stress, physical self-esteem, and quality of life [8].

One of the most important things college students can do for themselves is work on their physical self-esteem, which has been shown to have a major impact on their levels of motivation, persistence, and overall learning experience. Consequently, there may be a robust causal link between accomplishment in the physical domain and physical self-esteem [2]. On the other hand, it is not entirely clear from the previous research whether or not a connection between physical activity and higher self-esteem is associated with improved learning experience.

**Figure 1:**
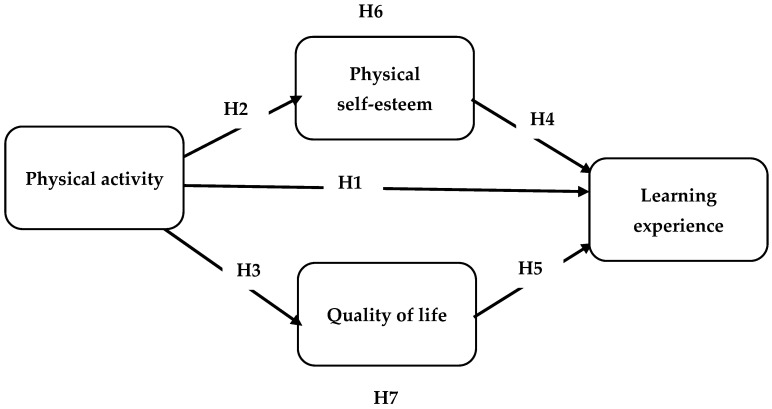
The research framework.

On the other hand, the negative health effects of physically being inactive are numerous and include an increased risk of diabetes, stroke, hypertension, and coronary heart disease [9]. According to the World Health Organization (2018), physical inactivity is the fourth leading risk factor for global mortality, accounting for an estimated 3,2 million deaths annually. In addition to lowering the risk of developing chronic diseases, engaging in regular physical activity (PA) can also boost QOL (Centers for Disease Control and Prevention) [10]. According to the World Health Organization Quality of Life Assessment Group, the term “quality of life” (QOL) is used to describe an individual’s internal evaluation of their own experiences, both good and bad [11,12,13], and includes both mental and physical aspects that influence one’s sense of contentment with life [4,14]. Positive correlations between PA and several quality-of-life indicators have been reported in previous research [15,16,17,18,19,20,21,22,23]. However, many of these studies have relied on the participation of the elderly [17,24,25] or those suffering from ongoing health issues [26,27,28]. In healthy young adults and college students, the connection between physical activity and its consequences (i.e., learning experience, QOL, and self-esteem) has been the subject of few published studies [19,29,30,31,32,33,34,35]. As a result, there is a dearth of data on the mental (quality of life and learning experience) and physical (physical self-esteem) outcomes associated with different PA levels among young adults and college students. There is a special interest in the college population because of the unique challenges that this cohort of young adults faces as they make the transition from high school to college [36,37], encountering greater accountability, competition, academic pressure, and time management demands [38,39,40]. Decreased physical activity (PA), expanded emotional and psychological stress, and sleep problems are all possible side effects of this change [30,36], as well as the onset of risky health behaviors such as drug and alcohol abuse [41], which may have an adverse effect on learning experience and quality of life. Therefore, more study is needed to determine what buffering factors, if any, may help sustainably improve college students’ learning experience and QOL. The current study investigates the impact of PA on LE through the mediating role of QOL and PSE. The study proposes five direct hypotheses and two indirect hypotheses (as shown in Figure 1), presented below.

**H1:**  *Physical activity has a positive and significant relationship with learning experience.*

**H2:**  *Physical activity has a positive and significant relationship with physical self-esteem.*

**H3:**  *Physical activity has a positive and significant relationship with quality of life.*

**H4:**  *Physical self-esteem has a positive and significant relationship with learning experience.*

**H5:**  *Quality of life has a positive and significant relationship with learning experience.*

**H6:**  *Physical self-esteem mediates the relationship between physical activity and learning experience.*

**H7:**  *Quality of life mediates the relationship between physical activity and learning experience.*

## 2. Materials and Methods

### 2.1. Study Design and Sampling

Data for a cross-sectional survey of university students in the Kingdom of Saudi Arabia (KSA) were collected from 1 October 2021 to 20 November 2021. As of 2020, Statista estimates that there will be nearly 1,000,000 college students attending 20 different universities in KSA. This study focused on public universities that provide access to free fitness centers and other facilities on the university campus. The study utilized an online self-reported survey made with Google Forms, and the link to the survey was distributed to students enrolled in KSA public universities through the personal networks of researchers (i.e., university lecturers) at various KSA public universities. They were asked to send the link to the survey to the students who were enrolled in their undergraduate programs via WhatsApp or email. The students were informed that their responses would be kept anonymous, and they were given the option to either accept or refuse to take part in the survey. It was up to the students whether they wanted to share their names, ages, genders, colleges, or universities. A total of 550 questionnaires were distributed, 520 collected, and 10 were discarded due to insufficient data, which resulted in a response rate of around 92% with 510 valid questionnaires.

Participants were randomly selected and given the opportunity to take part in the study by completing a structured questionnaire online and posting their responses on various online university-specific platforms. The survey targeted students who were not enrolled in any sports program as well as athletes who frequently participated in professional exercise or competed in national/international sports. The following were the criteria for exclusion: (a) age < 16 years, (b) use of illegal drugs on a regular basis, (c) cardiovascular or respiratory disease history, including diabetes, (d) pregnancy, and (e) former or current physical impairments that make even low-intensity exercise challenging.

An independent sample *t*-test method was conducted to assess the mean difference values for early and late answers. There were no statistically significant differences between early and late responses (*p* > 0.05), demonstrating that non-response bias is not an issue in this research.

### 2.2. Measures

The study survey has three main sections: the first one was designed as an introduction to state the study goals and some instructions for answering the questionnaires. The second part was constructed to provide data about the respondents themselves, involving their demographic characteristics. The third section contains the paper’s main survey with a five-point (1–5) Likert scale.

*Physical activity measure*. In order to measure regular exercise routines, we utilized the Godin Leisure Time Activity Scale [42]. We used a short, 3-item scale that explains self-reports of physical activity strength as strenuous “heart beats rapidly”, moderate “not exhausting”, and mild “minimal effort” exercise, taking into consideration a weeklong time frame during which the activity was carried out. We asked participants: “Considering 7 days (a week), during your leisure time, how often do you engage in any regular activity long enough to work up a sweat (heart beats rapidly)?” With responses being (5) Almost always, (4) Often, (3) Sometimes, (2) Seldom, and (1) Never. The items on the scale exhibited a high level of consistent reliability (a = 0.869).

*Physical self-esteem measure*. Physical self-esteem (PSE) was evaluated through the utilization of five items taken from the physical self-concept subscale of the Physical Self-Description Questionnaire (PSDQ; [43,44]). This scale offers a comprehensive assessment of the extent to which a person has favorable feelings about his or her physical self [43,45]. Students were asked to give answers to five questions (sample item: “I feel good about the way I look and what I can do physically”) on a 5-point Likert scale ranging from 1 (strongly disagree) to 5 (strongly agree). The reliability of the PSE5-items measure was determined to be adequate by Cronbach’s alpha (a) (a = 0.934).

*Learning experience measure*. The work conducted by MCGuire [10] served as the basis for the conceptualization and measurement of learning experience (LE). Students’ LE as an output of PA in the 2020–2021 first semester was self-reported. Sample items include “The use of physical activities has improved my comprehension of the concepts studied” and “The use of physical activities has allowed me to better understand the concepts studied”. Cronbach’s alpha (a) for the AP 3-item measure was adequate and satisfactory (a = 0.890).

*Quality of life measure*. QOL was measured using the Satisfaction with Life Scale (SWLS) [43], which consists of just five questions. The SWLS is a global cognitive assessment of an individual’s level of contentment with his or her life. Students were asked to rate how much they agreed with statements about their level of happiness. Sample items include: ‘‘The conditions of my life are excellent” and ‘‘If I could live my life over, I would change almost nothing”. The SWLS showed satisfactory reliability with high internal consistency and a Cronbach’s alpha (a) of a = 0.974.

Eleven professors and 15 senior students piloted the scale to ensure its reliability, simplicity, and clarity. As mentioned in the questionnaire’s opening, all data are anonymous and confidential. Since the research questionnaire relies on self-reporting, the likelihood of CMVis increased [46]. To test CMV, Harman’s single-factor analysis was performed with exploratory factor analysis (EFA), and all extracted factors were standardized to 1.0. Only one factor was extracted to explain 37% (less than 50%) of the variance in endogenous variables, ruling out the problem of CMV [47].

### 2.3. Data Analysis Techniques

We employed SPSS v25 for descriptive analysis (i.e., mean, standard deviation, skewness, and kurtoses). Consequently, the main data analysis technique to test the study’s proposed hypotheses was the Partial Least Squares Structural Equation Modeling (PLS-SEM), which was carried out using the SmartPLS4 program. PLS-SEM is a statistical method that sees widespread application in the field of management, where it is said to produce trustworthy results [48]. PLS-SEM is a non-parametric technique that takes advantage of the variance that can be explained in latent unobserved dimensions. Smart PLS-SEM requires less information about residual distributions, measurement scales, and sample sizes compared with the covariance-based SEM (COV–SEM) [49]. Smart PLS-SEM is thought to be appropriate for analyzing complex research models proposed as an estimation framework integrating related theories with empirical data. Following the suggestion of [50], a two-step approach was used, in which the adopted conceptual theoretical model first tested convergent and discriminant validity in the outer model, and then the inner model was evaluated to test the study hypotheses.

## 3. Data Analysis Findings

### 3.1. Demographic and Descriptive Statistics

In order to address the issue of missing data, SPSS version 25 determined the minimum value (1) and the maximum value (5). We discovered a few missing data points (less than 5%). As a consequence of this, the problem with the missing data was not a problem, and the outcomes of any solution would be the same [51]. The mean (M) values of the students’ responses were between 2.65 and 3.37, similarly, the standard deviation (S.D.) reading was between 0.87 and 1.28; these results indicate that the results are more dispersed and less condensed around the mean value [52]. Normal univariate normality was suggested by the findings of the readings for skewness and kurtosis (score distribution), which showed that there were no values larger than −2 or +2, respectively [53]. In addition, the VIF values for all of the study variables were found to be less than 0.5, which indicates that multi-collinearity is not an issue with our research [54].

Regarding demographics, the percentage of participating male students (80%) is significantly higher than the percentage of female students (20%). It was reasonable to discover that the vast majority of the students were younger than 25 years old (90%) (As shown in Table 1).

The students were enrolled in the College of Business Administration (28%), College of Computer Science and Information Technology (22%), College of Engineering (20), College of Education (17%), and College of Arts (13%). A total of 38% of the participating students were from King Faisal University, 35% belonged to Mohammad ibn Saud Islamic University, and finally 27% from Umm Al-Qura University. The majority (80%) of students practice physical activities inside the university, while 20% outside the university.

### 3.2. Evaluation Discriminant and Convergent Validity (Outer Measurement Model)

As suggested by Hair et al. [55], several criteria were evaluated to ensure the reliability and validity “discriminant and convergent” of the study measurement model “outer model”. This includes “internal consistency reliability” (Cronbach’s alpha), “composite reliability” (CR), “convergent validity”, and “discriminant validity”. Cronbach’s alpha (a) values ranged from 0.869 to 0.974, as shown in Table 2, and composite reliability (CR) values ranged from 0.875 to 0.974, giving evidence that that the study scale has an adequate level of internal reliability [51].

Second, the “Standardized Factor Loading” (SFL) values of each factor were greater than 0.70, providing additional evidence that the study dimensions have an adequate level of reliability. Thirdly, convergent validity was evaluated by determining if AVE values were greater than 0.5 [47]. This value represents the minimal level of acceptability to confirm convergent validity.

As suggested by Leguina [50], three additional criteria were employed to ensure the scale’s discriminant validity. The “cross-loading matrix,” the “Fornell–Larcker criterion method,” and the “heterotrait–monotrait method ratio” were among these criteria (HTMT). (1) According to what is shown in Table 3, in order to ensure the discriminant validity of the model, the outer-loading (bolded) of each variable needs to be higher than the cross-loading (with other measurements). (2) As is evident from looking at Table 4, the bolded diagonal AVE values are higher than the inter-variable correlation coefficient, which is evidence of a model with a high level of discriminant validity [47]. (3) According to Leguina [50], it is recommended that HTMT values be lower than 0.90. The HTMT levels in the study were significantly lower than the value used, as recommended (see Table 4). The previous results, when considered as a whole, confirm and provide support for the scale’s reliability, discriminant validity, and convergent validity, all of which were approved in the study’s outer measurement model. As a consequence of this, we are able to proceed with the structural outer model so that we can test the hypotheses of the study.

### 3.3. Assessment of the Study Hypotheses (Structural Inner Model)

An assessment using structural equations was carried out in order to test the hypotheses that were proposed for the study. To be more specific, the primary objective is to investigate whether or not the model is able to explain and forecast the variation in the endogenous variables that are brought about by the exogenous variable [55]. Furthermore, several criteria were employed as recommended by [55,56,57] to ensure the goodness of the model fit. These criteria include the R2 value (at least 0.10); “Stone–Geisser Q2 “(more than 0.0); NFI (more than 0.90), and the SRMR value (less than 0.08). As displayed in Table 5, all the endogenous dimensions showed adequate R2 and Q2 values: physical self-esteem (R2 = 0.176, Q2 = 0.043); quality of life (R2 = 0.449, Q2 = 0.444); and learning experience (R2 = 0.043, Q2 = 0.036), giving evidence that the study model appropriately represents the empirical data and has an acceptable predictive power [57]. Furthermore, the SRMR value (0.047) and the NFI value (0.911) supported the good model fit (GoF).

**Figure 2:**
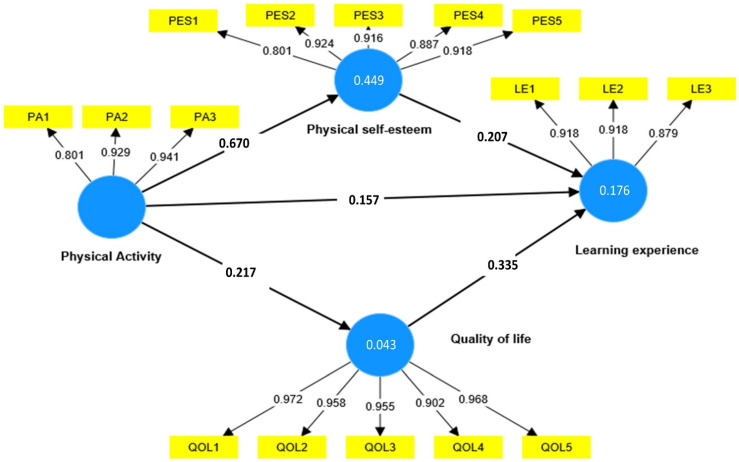
Structural and Measurement Model.

Finally, in smart PLS4, a bootstrapping approach was used to calculate the path coefficient with its attributed t-value for both direct and mediating interrelationships. The current study proposed seven hypotheses, five of which are direct relationships, and two are indirect. The results of smart PLS indicated that physical activity has a direct positive and significant impact on learning experience (β = 0.157, t-value = 5.565, *p* < 0.01); physical self-esteem (β = 0.670, t-value = 17.33, *p* < 0.01); and quality of life (β = 0.217, t-value = 4.163, *p* < 0.01), consequently, the H1, H2, and H3 hypotheses were supported. In return, physical self-esteem was found to have a positive and significant impact on learning experience (β = 0.207, t-value = 3.226, and *p* < 0.01), while the quality of life was found to have a positive and significant impact on learning experience (β = 0.335, t-value = 6.035, *p* < 0.01); therefore, hypotheses H4 and H5 were supported, as shown in Table 6 and Figure 2. In addition, the results provide information regarding the specific indirect effect in order to test the mediation effects of physical self-esteem and quality of life in the relationship between physical activity and learning experience. All specific indirect effects were found to be positive and statistically significant, supporting the mediating effects of physical self-esteem in the relationships between physical activity and learning experience (β = 0.072, t-value = 3,578, and *p* 0.01). Similarly, quality of life mediates the relationship between physical activity and learning experience (β = 0.139, t-value = 3.147, and *p* < 0.01), hence supporting hypotheses H6 and H7.

## 4. Discussion and Implications

This research examines four aspects of the relationships: (1) the direct relationship between PA and QOF and PSE and LE; (2) the direct effects of PSE and LE; (3) the effects of QOL on LE; and (4) the mediating role of PSE and QOL in the relationship between PA and LE. The findings of this study give support to the growing body of research that demonstrates consistent positive relationships between physical activity and learning experience [8,34,36]. The investigation of these relationships in greater depth, with the goal of determining whether or not this connection can be mediated by other aspects (i.e., QOL and PSE), was one of the primary goals of this study. Previous empirical research has found positive correlations between PA, LE, and several quality-of-life variables [15,17,19,21,23]. However, a significant number of these studies have depended on the participation of the elderly [17,24] or those who have been dealing with health problems for a long time [26,27]. Few published studies have investigated the relationship between healthy young adults and college students’ levels of physical activity and the outcomes of that activity (specifically, their learning experience, quality of life, and levels of self-esteem) [19,29,30,32,34]. Because of this, there is a paucity of information regarding the mental (quality of life and learning experience) and physical (physical self-esteem) outcomes that are associated with various levels of PA among young adults and college students. College students face unique challenges as they transition from high school to college, so they are of particular interest [36]. College students are frequently subjected to increased accountability, competition, academic pressure, and time management requirements [38,40]. This change may result in decreased physical activity (PA), increased emotional and psychological stress, and sleep problems [30,36], as well as the onset of potentially harmful health behaviors such as drug and alcohol abuse [41], which could have a negative impact on learning experience and quality of life. Therefore, there is a need for additional research to determine what buffering factors, if any, may help sustainably improve the learning experience and quality of life of college students. The current study investigates the impact of PA on LE through the mediating role of QOL and PSE.

The findings of this study supported the positive direct impact of PA on QOL, PSE, and LE. These findings are consistent with previous research indicating that one way to combat procrastination is to engage in routines and activities on a consistent basis that call for a predetermined amount of time at regular intervals [17,19,36]. Therefore, greater dedication to PA is associated with both increased and improved time management. This makes sense, given that research suggests that people who spend more time on PA are less likely to put things off in their everyday lives. These results and their assessment show a previously unknown benefit of PA. To be more specific, the advantages of PA can be broken down into three categories: an improvement in one’s sense of self-esteem; an enhancement of one’s learning experience; and a marked improvement in one’s quality of life.

Additionally, the findings demonstrated that the PSE significantly increased in the participants who engaged in consistent routines of physical activity. The findings of this study are consistent with those obtained by other researchers [10,15,32,34,36]. Generally, a person’s success and achievement are determined by his or her sense of self-worth and self-esteem. Self-esteem is one of the fundamental requirements of human life. People need to have a healthy and worthy perception of themselves on all levels, including intellectually, emotionally, and physically. This emotion serves the purpose of motivating a person to succeed in the tasks that they have set for themselves in life, including engaging in physical activity, which is closely related to one’s self-esteem and is something that is being addressed by major education organizations.

Furthermore, the study findings highlighted the significant impact of PA on LE. This result is in line with [8,15,17,34,36,38]. The findings of the present study suggest that PA is essential to maximize student LE. PA can promote LE, with just a single exercise of moderate PA having a significant impact on students’ ability to comprehend theoretical concepts and generate a better learning environment, significantly improving in participants with a high level of physical self-esteem and quality of life. QOL was found to have a positive significant impact on LE. This result is consistent with previous research indicating that high levels of QOL correlate with academic success [58,59,60]. These findings demonstrate the importance of QOL to positive educational progress. Few studies have examined the relationship between QOL and actual learning experience, despite the fact that there is a strong relationship between QOL and student behavior and perceived academic competence. High levels of QOL appear to have a positive effect on learning experience, which in turn improves future QOL. Students may perform well in their college because they are generally happy, and good grades may contribute to that happiness.

Many college students’ self-esteem declines during adolescence. The results of this study indicate that involvement in physical activities may aid some adolescents in navigating this challenging period and improve their learning experience. Finally, the study highlighted the mediation effect of QOL in improving the relationship between PA and learning experience. High levels of PA appear to exert a positive influence on QOL, which in turn boosts future learning experience. Simply put, students may do well in college because they are generally happy with their lives and have high physical self-esteem.

The study has some academic and managerial implications, as sport fitness initiatives in universities may be beneficial on three levels: as a vehicle for satisfaction and fulfillment, as a treatment for psychiatric disorders, and as a facilitator of learning and academic achievement. While the majority of attempts to improve learning experience are focused on study skills and teaching methods, this study gives credibility to the significance of enhancing academic achievement through PA. This study’s findings indicate that PA, PSE, and QOL do not hinder a good learning experience (or vice versa) but rather promote better learning. According to the Noddings [61], who states that “happiness and education are properly, intimately related”, it would appear that students retain information more effectively when they are happy and have a high level of physical self-esteem. Policy makers as well are advised to provide free sport exercise facilities to help students practice physical activities during their academic life; professors and university leaders are required as well to make changes in curricula to leave enough time for students between lectures to be able to practice physical activities.

The study has a number of limitations, most of which are anticipated to be overcome by subsequent investigation. This study tested the mediating effects of PSE and QOL in the relationship between PA and LE. However, several other factors such as gender, family support, and year of study may act as moderators that can affect the tested relationships. It is strongly recommended that the authors expand the scope of this research in the future by investigating a wider range of factors that have an effect on the LE. In addition, because these data are cross-sectional, it is not possible to determine the specific causal relationships that exist between the variables. Moreover, despite the fact that we worked hard to steer clear of CMV in accordance with Podsakoff’s [62] suggestions, in the future, researchers may choose to support the proposed model of this study with either longitudinal data or a combination of data sources. Finally, by utilizing a multi-group analysis technique, the suggested research model may be tested in a different context (country and/or industry), and results can be compared to verify or falsify the current study results [63].

## 5. Conclusions

The study investigated the direct impact of PA on PSE, QOL, and LE, as well as the indirect impact through the mediating role of PSE and QOL in the relationship between PA and LE. Data were collected through a self-reported online questionnaire, and 510 students completed the study survey. The obtained data were analyzed through PLS-SEM with the SmartPLS 4 program. The results support the direct impacts of PA on PSE, QOF, and LE. Additionally, QOL and PSE were found to act as a mediator that strengthens the relationship between PA and LE. Additional studies can use some moderating variables (i.e., gender, student year of study) and conduct a multi-group analysis to test the proposed model in two different groups or a different context (population, industry, or country).

## Figures and Tables

**Table 1:** Demographic and Descriptive Statistics.

Variable		n (%)
**Total sample**		n = 510
**Gender**	Male	408 (80%)
	Female	102 (20%)
**Age Group**	17–20	306 (60%)
	21–25	153(30%)
	Above 25	51 (10%)
**College**	College of Arts	66 (13%)
	College of education	87 (17%)
	College of business administration	143 (28%)
	College of computer science and information technology	112 (22%)
	College of engineering	102 (20%)
**University**	King Faisal University	194 (38%)
	Imam Mohammad ibn Saud Islamic University	179 (35%)
	Umm Al-Qura University	137 (27%)
**Exercises inside the university/outside the university**	Inside	408 (80%)
	Outside	102 (20%)

**Table 2:** Evaluation of the Outer Measurement Model (*a*, C.R, and AVE) and multi-collinearity (VIF), M, S.D, Skewness, and Kurtoses values.

Abbr.		Outer Loading	α	C.R	AVE	VIF	M	S.D	Skeweness	Kurtoses
Learning Experience			0.890	0.901	0.819					
LE1	“The use of physical activities has improved my comprehension of the concepts studied”.	0.918				2.709	2.70	1.32	0.472	−0.999
LE2	“The use of physical activities has led to a better learning experience in my study”.	0.918				3.090	2.67	1.28	0.528	−0.891
LE3	“The use of physical activities has allowed me to better understand the concepts studied”.	0.879				2.308	2.65	1.28	0.560	−0.849
Physical self-esteem			0.934	0.942	0.793					
PSE1	“Physically, I am happy with myself”.	0.801				2.277	3.16	1.03	0.144	−0.0538
PSE2	“Physically, I feel good about myself”.	0.924				4.572	2.88	1.09	0.499	−0.846
PSE3	“I feel good about who I am and what I can do physically”.	0.916				4.414	2.89	1.18	0.375	−0.990
PSE4	“I feel good about who I am physical”.	0.887				3.529	2.98	1.10	0.393	−1.045
PSE5	“I feel good about the way I look and what I can do physically”.	0.918				4.183	2.81	1.25	0.484	−0.963
Quality of life			0.974	0.974	0.905					
QOL1	“In most ways my life is ideal”.	0.972				4.846	3.18	0.87	−0.201	0.422
QOL2	“I am satisfied with my life”.	0.958				4.113	3.18	0.87	−0.0204	0.419
QOL3	“The conditions of my life are excellent”.	0.955				4.073	3.17	0.89	−0.207	0.358
QOL4	“So far, I have gotten the important things I want in life”.	0.902				4.535	3.02	1.03	−0.385	−0.085
QOL5	“If I could live my life over, I would change almost nothing”.	0.968				4.407	3.16	0.88	−0.140	0.280
Physical Activity			0.869	0.875	0.796					
PA1	“Considering 7 days (a week), during your leisure time, how often do you engage in any regular activity long enough to work up a sweat (heart beats rapidly)?” (i.e., Carrying heavy loads, soccer game, or bicycling fast 14–16 mph)”.	0.801				1.573	2.82	1.100	0.478	−0.738
PA2	“Considering 7 days (a week), during your leisure time, how often do you engage in any regular activity that can be considered as moderate activity i.e., walking very brisk, bicycling light effort (10–12 mph)”.	0.929				4.213	3.37	0.951	−0.113	−0.170
PA3	“Considering 7 days (a week), during your leisure time, how often do you engage in any regular activity that can be considered as mild activity i.e., walking slowly, sitting using computer, fishing sitting”.	0.941				4.500	3.35	0.985	0.203	−0.154

*a*: Cronbach’s alpha; C.R: composite reliability; AVE: average variance extracted, VIF: variance inflation factor.

**Table 3:** Cross-loading for study factors.

	Learning Experience	Physical Activity	Physical Self-Esteem	Quality of Life
LE1	**0.962**	0.066	0.095	0.265
LE2	**0.797**	0.052	0.051	0.300
LE3	**0.798**	0.140	0.070	0.273
PA1	0.114	**0.784**	0.583	0.145
PA2	0.022	**0.836**	0.624	0.188
PA3	0.111	**0.880**	0.638	0.224
PES1	0.036	0.525	**0.712**	0.276
PES2	0.028	0.648	**0.878**	0.343
PES3	0.077	0.712	**0.966**	0.340
PES4	0.126	0.631	**0.854**	0.186
PES5	0.008	0.652	**0.884**	0.254
QOL1	0.303	0.205	0.335	**0.928**
QOL2	0.292	0.213	0.341	**0.918**
QOL3	0.311	0.221	0.334	**0.969**
QOL4	0.302	0.191	0.196	**0.907**
QOL5	0.312	0.220	0.317	**0.970**

**Table 4:** Fornell–Larker Criterion and HTMT results.

	Fornell–Larcker Criterion				HTMT Results			
	1	2	3	4	1	2	3	4
1-Learning experience	**0.905**							
2-Physical Activity	0.088	**0.892**			0.108			
3-Physical self-esteem	0.002	0.670	**0.890**		0.114	0.740		
4-Quality of life	0.303	0.207	0.311	**0.951**	0.327	0.223	0.325	

**Table 5:** Coefficient of determination (R2) and (Q2) and model fit (SRMR-NFI).

Endogenous Latent Factors	(R^2^)	(Q^2^)
Physical self-esteem	0.176	0.043
Quality of life	0.449	0.444
Learning Experience	0.043	0.036
**Model Fit**	**SRMR**	**NFI**
	0.047	0.911

**Table 6:** Study Hypotheses Results.

	Hypotheses	Beta (β)	(T-Value)	*p* Values	Results
H1	Physical Activity → Learning experience	0.157	2.565	0.010	Accepted
H2	Physical Activity → Physical self-esteem	0.670	17.33	0.000	Accepted
H3	Physical Activity → Quality of life	0.217	4.163	0.000	Accepted
H4	Physical self-esteem → Learning experience	0.207	3.226	0.001	Accepted
H5	Quality of life → Learning experience	0.335	6.035	0.000	Accepted
H6	Physical Activity → Quality of life → Learning experience	0.072	3.578	0.000	Accepted
H7	Physical Activity → Physical self-esteem → Learning experience	0.139	3.174	0.002	Accepted

## Data Availability

Data are available upon request from researchers who meet the eligibility criteria. Kindly contact the first author privately through e-mail.
